# Supplementary material for: Analysis of PPARγ Signaling Activity in Psoriasis
Source: Int J Mol Sci. 2021 Aug 10;22(16):8603. doi: 10.3390/ijms22168603 (PMC8395241; doi:10.3390/ijms22168603)
Supplement: Supplementary file 1 [file ijms-22-08603-s001.zip › Supplemental materials_Analysis of PPARg signaling activity in psoriasis/Pathway models/Models images and html files/Model of PPARG related pathways in psoriasis (short version)/14332.html]

NLRP3


# Protein NLRP3

|  |  |
| --- | --- |
| URN | urn:agi-llid:9558 |
| Total Entities | 0 |
| Connectivity | 4753 |
| Name | NLRP3 |
| Description | NLR family, pyrin domain containing 3 |
| Notes | This gene encodes a pyrin-like protein containing a pyrin domain, a nucleotide-binding site (NBS) domain, and a leucine-rich repeat (LRR) motif. This protein interacts with the apoptosis-associated speck-like protein PYCARD/ASC, which contains a caspase recruitment domain, and is a member of the NALP3 inflammasome complex. This complex functions as an upstream activator of NF-kappaB signaling, and it plays a role in the regulation of inflammation, the immune response, and apoptosis. Mutations in this gene are associated with familial cold autoinflammatory syndrome (FCAS), Muckle-Wells syndrome (MWS), chronic infantile neurological cutaneous and articular (CINCA) syndrome, and neonatal-onset multisystem inflammatory disease (NOMID). Multiple alternatively spliced transcript variants encoding distinct isoforms have been identified for this gene. Alternative 5' UTR structures are suggested by available data; however, insufficient evidence is available to determine if all of the represented 5' UTR splice patterns are biologically valid. [provided by RefSeq, Oct 2008] |
| ObjectType | Protein |

---

|  |  |
| --- | --- |
| Pathway | NOD-like Receptors |
|  | Dectin-1 (CLEC7A) Signaling |
|  | Toll-like Receptor Independent Sterile Inflammation |
|  | Proteins Involved in Myocarditis |
|  | Inflammatory Reaction in Acne Vulgaris |
|  | Apoptotic Keratinocytes Clearance Recession in Systemic Lupus Erythematosus |
|  | Dendritic Cell Activation in Systemic Lupus Erythematosis |
|  | Plasmacytoid Dendritic Cell in Diabetes Mellitus Type 1 |
|  | Macrophage Function in Diabetes Mellitus Type 1 |
|  | Conventional Dendritic Cell Role in Diabetes Mellitus Type 1 |
|  | Proteins Involved in non-Alcoholic Fatty Liver Disease |
|  | Crohn's Disease |
|  | Macrophage Dysfunction |
|  | Dendritic Cell Function in Ulcerative Colitis |
|  | Epithelial Cell in the Innate Immune Response in Ulcerative Colitis |
|  | Antigen-Presenting Cell Role in Asthma |
|  | Proteins Involved in Gout |
|  | Monocytes Function in Gout |
|  | Dendritic Cell Dysfunction in Psoriatic Arthritis |
|  | Proteins Involved in Periodontitis |
|  | PAPA Syndrome |
|  | Blau Syndrome |
|  | Cryopyrin (NLPR3) Associated Periodic Syndromes |
|  | Familial Cold Autoinflammatory Syndrome 2 |
|  | Familial Mediterranean Fever |
|  | Majeed Syndrome |
|  | Proteins Involved in Age-Related Macular Degeneration |
|  | Immune System Role in Otitis Media |
|  | Alveolar Macrophages Dysfunction in COPD |
|  | Elevated Receptors -> Expression Targets in Mouth |
|  | Asbestosis- and Silicosis-Associated Inflammation Provokes Cancer Development |
|  | Proteins/Ions Transporters -> Expression Targets in Lymphoid System and Blood |
|  | P2RX7 -> Expression Targets in Nerve Tissue |
|  | e |
|  | pathway 4 |
|  | pprarg neg, uknown targets, ps-positive |
|  | Model of PPARG signaling in psoriasis |
|  | Model of PPARG related pathways in psoriasis (short version) |
|  | sars-cov binding |
|  | sars-cov no binding |
|  | Dendritic Cell Activation in COVID19 |
|  | Overview. Diseases of the digestive system (chapter 10) |
|  | 1\_Inflammation in keratinocytes in the pathology of acne |
|  | 1\_Insufficient activation of immune response in the middle ear epithelium cells in otitis media |
|  | 3\_Role of alveolar macrophages in pulmonary tissue destruction in COPD |
|  | 1\_Th2 cell response in asthma |
|  | 2\_Paneth cell dysfunction in Crohn's disease |
|  | 1\_Defects in response to pathogens in the gut promotes inflammation in Crohn's disease |
|  | 2\_Intestine epithelial cells drive immunological responses in ulcerative colitis |
|  | 4\_1\_Polymorphisms associated with inflammatory bowel diseases |
|  | 1\_Antigen-presenting cells promote islet dysfunction and immune system activation in T1D |
|  | Figure 4\_Diabetes mellitus Type 1: conventional dendritic cell |
|  | drug-target pairs direct regulation |

---

|  |  |
| --- | --- |
| Group | Genes with Mutations Associated with Vitiligo |
|  | binding partnetrs for SARSs |
|  | other partners for SARS |

---

|  |  |
| --- | --- |
| MedScan ID | 9558 |

---

|  |  |
| --- | --- |
| LocusLink ID | 9558 |
|  | 114548 |
|  | 216799 |
|  | 287362 |

---

|  |  |
| --- | --- |
| Alias | deafness, autosomal dominant 34 |
|  | OTTHUMP00000038410 |
|  | AII/AVPs |
|  | NACHT, LRR and PYD containing protein 3 |
|  | CLR1.1 |
|  | Familial cold autoinflammatory syndrome gene |
|  | FCAS1 |
|  | PYRIN-containing APAF1-like protein I |
|  | C1orf7 |
|  | Muckle-Wells syndrome protein |
|  | neuronal apoptosis inhibitor protein 3 |
|  | Muckle-Wells syndrome gene |
|  | chromosome 1 open reading frame VII |
|  | NACHT domain-, leucine-rich repeat-, and PYD-containing protein 3 |
|  | RP23-210M6.4 |
|  | cold-induced autoinflammatory syndrome 1 protein |
|  | familial cold urticaria gene |
|  | KEFH |
|  | FLJ95925 |
|  | NALP3 |
|  | nucleotide-binding oligomerization domain, leucine rich repeat and pyrin domain containing 3 |
|  | Mmig1 |
|  | NACHT, LRR and PYD containing protein III |
|  | NLR family, pyrin domain containing 3 |
|  | AGTAVPRL |
|  | MWS |
|  | OTTMUSP00000005970 |
|  | cryopyrin, NACHT, LRR and PYD domains-containing protein 3 |
|  | PYPAF I |
|  | cryopyrin |
|  | OTTHUMP00000038408 |
|  | NACHT, LRR and PYD domains-containing protein 3 |
|  | mast cell maturation-associated-inducible protein 1 |
|  | mast cell maturation inducible protein 1 |
|  | NACHT domain leucine rich repeat and pyrin domain containing protein 3 |
|  | chromosome 1 open reading frame 7 |
|  | cold autoinflammatory syndrome 1 homolog |
|  | AII/AVP |
|  | cold induced autoinflammatory syndrome 1 protein |
|  | OTTHUMP00000038411 |
|  | cold-induced autoinflammatory syndrome-1 protein |
|  | DFNA34 |
|  | cold autoinflammatory syndrome 1 |
|  | OTTHUMP00000038409 |
|  | PYRIN-containing APAF1-like protein 1 |
|  | angiotensin/vasopressin receptor AII/AVP-like |
|  | caterpiller protein 1.1 |
|  | FCAS-1 |
|  | MGC129375 |
|  | PYPAF1 |
|  | aii/avp receptor-like |
|  | NLRP3 |
|  | cold autoinflammatory syndrome I |
|  | CIAS1 |
|  | neuronal apoptosis inhibitor protein III |
|  | NACHT/LRR/pyrin domain-containing protein 3 |
|  | Mmig I |
|  | CIAS I |
|  | AII |
|  | AVP |
|  | FCU |
|  | FCAS |
|  | cold autoinflammatory syndrome 1 protein |
|  | cryopyrin, NACHT, LRR and PYD domains - containing protein 3 |
|  | cold autoinflammatory syndrome 1 protein homolog |

---

|  |  |
| --- | --- |
| GO ID | 0005524 |
|  | 0042834 |
|  | 0043565 |
|  | 0008134 |
|  | 0044546 |
|  | 0006919 |
|  | 0006915 |
|  | 0071222 |
|  | 0071224 |
|  | 0002374 |
|  | 0006952 |
|  | 0050830 |
|  | 0051607 |
|  | 0009595 |
|  | 0006954 |
|  | 0045087 |
|  | 0032611 |
|  | 0050701 |
|  | 0032621 |
|  | 0032088 |
|  | 1901223 |
|  | 0002674 |
|  | 0050728 |
|  | 0050713 |
|  | 0051092 |
|  | 2000321 |
|  | 2000553 |
|  | 0045630 |
|  | 0043280 |
|  | 0050718 |
|  | 0032736 |
|  | 0032753 |
|  | 0032754 |
|  | 0045944 |
|  | 0002830 |
|  | 0051259 |
|  | 0016579 |
|  | 0007165 |
|  | 0000139 |
|  | 0072559 |
|  | 0005737 |
|  | 0005829 |
|  | 0005783 |
|  | 0005576 |
|  | 0005634 |
|  | 0000166 |
|  | 0002376 |
|  | 0050727 |
|  | 0061702 |
|  | 0005623 |
|  | 0006917 |
|  | 0035872 |
|  | 0006351 |
|  | 0006355 |
|  | 0005515 |
|  | 0042347 |
|  | 0009617 |

---

|  |  |
| --- | --- |
| KEGG ID | rno:287362 |
|  | hsa:114548 |
|  | mmu:216799 |

---

|  |  |
| --- | --- |
| Organism | Homo sapiens {Organism urn:agi-taxid:9606} |
|  | Mus musculus {Organism urn:agi-taxid:10090} |
|  | Rattus norvegicus {Organism urn:agi-taxid:10116} |
|  | Homo sapiens |
|  | Mus musculus |
|  | Rattus norvegicus |

---

|  |  |
| --- | --- |
| Mouse chromosome position | 11 |

---

|  |  |
| --- | --- |
| OMIM ID | 606416 |
|  | 607115 |
|  | 617772 |
|  | 191900 |
|  | 120100 |
|  | 148200 |

---

|  |  |
| --- | --- |
| Rat chromosome position | 10q22 |

---

|  |  |
| --- | --- |
| Hugo ID | 16400 |
|  | HGNC:16400 |

---

|  |  |
| --- | --- |
| Human chromosome position | 1q44 |

---

|  |  |
| --- | --- |
| Swiss-Prot Accession | Q96P20 |
|  | Q96P20.3 |
|  | Q8R4B8 |
|  | Q8R4B8.1 |
|  | D4A523 |
|  | D4A523.2 |
|  | A0A024R5Q0 |
|  | B2RC97 |
|  | B7ZKS9 |
|  | B7ZKT2 |
|  | B7ZKT3 |
|  | O75434 |
|  | Q17RS2 |
|  | Q59H68 |
|  | Q5JQS8 |
|  | Q5JQS9 |
|  | Q6TG35 |
|  | Q8TCW0 |
|  | Q8TEU9 |
|  | Q8WXH9 |
|  | Q1JQ87 |
|  | Q1JQ88 |
|  | Q6JEL0 |
|  | T1W2H6 |
|  | Q65Z67 |
|  | Q5SXI6 |

---

|  |  |
| --- | --- |
| GenBank ID | NC\_000001 |
|  | NM\_001127461 |
|  | NP\_001120933 |
|  | NM\_001079821 |
|  | NP\_001073289 |
|  | XM\_017000183 |
|  | XP\_016855672 |
|  | XM\_017000184 |
|  | XP\_016855673 |
|  | XM\_017000182 |
|  | XP\_016855671 |
|  | XM\_017000181 |
|  | XP\_016855670 |
|  | XM\_011544048 |
|  | XP\_011542350 |
|  | XM\_024452862 |
|  | XP\_024308630 |
|  | XM\_024452874 |
|  | XP\_024308642 |
|  | NM\_004895 |
|  | NP\_004886 |
|  | NM\_001243133 |
|  | NP\_001230062 |
|  | NM\_001127462 |
|  | NP\_001120934 |
|  | NM\_183395 |
|  | NP\_899632 |
|  | NG\_007509 |
|  | AB120959 |
|  | BAD51451 |
|  | AB120960 |
|  | BAD51452 |
|  | AB120961 |
|  | BAD51453 |
|  | AB120962 |
|  | BAD51454 |
|  | AC104335 |
|  | AH011140 |
|  | AAL12497 |
|  | AAL12498 |
|  | AL606804 |
|  | CH471148 |
|  | EAW77183 |
|  | EAW77184 |
|  | EAW77185 |
|  | EAW77186 |
|  | EAW77187 |
|  | MG557564 |
|  | AXK59206 |
|  | AB208891 |
|  | BAD92128 |
|  | AF054176 |
|  | AAC39910 |
|  | AF410477 |
|  | AAL33908 |
|  | AF418985 |
|  | AAL14640 |
|  | AF420469 |
|  | AAL65136 |
|  | AF427617 |
|  | AAL33911 |
|  | AF468522 |
|  | AAL78632 |
|  | AK027194 |
|  | AK314998 |
|  | BAG37494 |
|  | AL601856 |
|  | AY092033 |
|  | AAM14669 |
|  | AY422168 |
|  | AAQ98889 |
|  | BC117211 |
|  | AAI17212 |
|  | BC143359 |
|  | AAI43360 |
|  | BC143362 |
|  | AAI43363 |
|  | BC143363 |
|  | AAI43364 |
|  | DA244290 |
|  | Q96P20 |
|  | NC\_000077 |
|  | XM\_006532857 |
|  | XP\_006532920 |
|  | XR\_388400 |
|  | XM\_006532858 |
|  | XP\_006532921 |
|  | NM\_145827 |
|  | NP\_665826 |
|  | NM\_001359638 |
|  | NR\_153314 |
|  | AH013321 |
|  | AAR03541 |
|  | AH013322 |
|  | AAR03542 |
|  | AH013323 |
|  | AAR03543 |
|  | AL592522 |
|  | AF486632 |
|  | AAL90874 |
|  | AW121591 |
|  | AY337285 |
|  | AAR03540 |
|  | AY355340 |
|  | AAR14737 |
|  | AY495376 |
|  | AAS75794 |
|  | AY495377 |
|  | AAS75795 |
|  | BC116174 |
|  | AAI16175 |
|  | BC116175 |
|  | AAI16176 |
|  | BY186774 |
|  | BY722759 |
|  | KF032621 |
|  | AGU01502 |
|  | Q8R4B8 |
|  | NC\_005109 |
|  | XM\_017597078 |
|  | XP\_017452567 |
|  | XM\_006246453 |
|  | XP\_006246515 |
|  | XM\_006246458 |
|  | XP\_006246520 |
|  | XM\_006246455 |
|  | XP\_006246517 |
|  | XM\_006246457 |
|  | XP\_006246519 |
|  | NM\_001191642 |
|  | NP\_001178571 |
|  | AC\_000078 |
|  | AABR07029714 |
|  | AABR07029715 |
|  | AABR07072123 |
|  | AAHX01064203 |
|  | AAHX01064204 |
|  | AC123316 |
|  | FQ231619 |
|  | D4A523 |
|  | CAI17154 |
|  | CAI17153 |
|  | CAI17155 |
|  | XP\_006246518 |
|  | XP\_006246516 |
|  | AY337302 |
|  | AY337303 |
|  | AY337300 |
|  | AY337301 |
|  | AY337306 |
|  | AY337304 |
|  | AY337305 |
|  | NW\_001071914 |
|  | AAHY01147154 |
|  | CAI24551 |
|  | CAI24552 |
|  | XM\_006711733 |
|  | XM\_005273036 |
|  | XM\_005273037 |
|  | XM\_005273038 |
|  | NC\_018912 |
|  | XP\_006711796 |
|  | AY051113 |
|  | AY051112 |
|  | XM\_006246454 |
|  | XM\_006246456 |
|  | XP\_005273093 |
|  | XP\_005273094 |
|  | XP\_005273095 |
|  | AY051115 |
|  | AY051114 |
|  | AY051117 |
|  | AY051116 |
|  | XM\_011544049 |
|  | XP\_006532922 |
|  | XM\_011544051 |
|  | XM\_011544050 |
|  | XM\_011544053 |
|  | XM\_011544052 |
|  | XM\_011544055 |
|  | XM\_011544054 |
|  | AMYH02003133 |
|  | AMYH02003132 |
|  | AY056060 |
|  | AY337289 |
|  | AY337287 |
|  | AY337288 |
|  | AY337286 |
|  | AABR06064074 |
|  | AABR06064075 |
|  | XP\_011542356 |
|  | XP\_011542357 |
|  | XP\_011542354 |
|  | XP\_011542355 |
|  | XP\_011542352 |
|  | XP\_011542353 |
|  | XP\_011542351 |
|  | AY056059 |
|  | AC\_000133 |
|  | XM\_006532859 |
|  | AY337294 |
|  | AY337295 |
|  | AY337292 |
|  | AY337293 |
|  | AY337290 |
|  | AY337291 |
|  | ABBA01031787 |
|  | ABBA01031788 |
|  | AY337298 |
|  | AY337299 |
|  | AY337296 |
|  | AY337297 |
|  | NT\_167186 |
|  | NW\_001838554 |
|  | NW\_927572 |
|  | AC\_000044 |
|  | CR609473 |
|  | Q65Z67 |
|  | NT\_096135 |
|  | Q5SXI6 |
|  | XM\_220513 |
|  | XP\_220513 |
|  | NW\_047334 |
|  | NW\_001084656 |
|  | XM\_001075398 |
|  | XP\_001075398 |

---

|  |  |
| --- | --- |
| Swiss-Prot ID | NLRP3\_RAT |
|  | NLRP3\_HUMAN |
|  | NLRP3\_MOUSE |
|  | NALP3\_HUMAN |
|  | NALP3\_MOUSE |
|  | B2RC97\_HUMAN |
|  | B7ZKT2\_HUMAN |
|  | B7ZKT3\_HUMAN |

---

|  |  |
| --- | --- |
| Cell Localization | Cytoplasm |
|  | Cytosol |
|  | Inflammasome |
|  | Endoplasmic reticulum |
|  | Secreted |
|  | Nucleus |
|  | Golgi apparatus membrane |
|  | Cytoplasm, cytosol |

---

|  |  |
| --- | --- |
| Ensembl ID | ENSG00000162711 |
|  | ENSP00000375704.3 |
|  | ENST00000391828.7 |
|  | ENSP00000355453.2 |
|  | ENST00000366497.6 |
|  | ENSP00000375703.2 |
|  | ENST00000391827.2 |
|  | ENSP00000337383.3 |
|  | ENST00000336119.7 |
|  | ENSP00000294752.3 |
|  | ENST00000348069.6 |
|  | ENSMUSG00000032691 |
|  | ENSMUSP00000098707.2 |
|  | ENSMUST00000101148.8 |
|  | ENSMUSP00000078440.3 |
|  | ENSMUST00000079476.9 |
|  | ENSRNOG00000003170 |
|  | ENSRNOP00000004280.3 |
|  | ENSRNOT00000004280.5 |
|  | ENSP00000375704 |
|  | ENSMUST00000079476 |
|  | ENSP00000375703 |
|  | ENST00000366497 |
|  | ENSMUSP00000098707 |
|  | ENSP00000355453 |
|  | ENST00000348069 |
|  | ENST00000336119 |
|  | ENST00000391828 |
|  | ENST00000391827 |
|  | ENSP00000337383 |
|  | ENSMUSP00000078440 |
|  | ENSRNOT00000004280 |
|  | ENSRNOP00000004280 |
|  | ENSP00000294752 |
|  | ENSMUST00000101148 |

---

|  |  |
| --- | --- |
| MGI ID | MGI:2653833 |
|  | 2653833 |

---

|  |  |
| --- | --- |
| RGD ID | 1308314 |

---

|  |  |
| --- | --- |
| Unigene ID | Mm.54174 |
|  | Hs.159483 |
|  | Rn.214177 |

---

|  |  |
| --- | --- |
| Homologene ID | 3600 |

---

|  |  |
| --- | --- |
| IPI ID | IPI00374129 |
|  | IPI00153873 |
|  | IPI00556137 |
|  | IPI00742837 |
|  | IPI00620897 |
|  | IPI00553033 |
|  | IPI00451585 |
|  | IPI00621784 |
|  | IPI00620168 |
|  | IPI00198971 |
|  | IPI00777914 |
|  | IPI00777199 |

---
